# Immunomodulatory Function of Polyvinylpyrrolidone (PVP)-Functionalized Gold Nanoparticles in *Vibrio*-Stimulated Sea Urchin Immune Cells

**DOI:** 10.3390/nano11102646

**Published:** 2021-10-08

**Authors:** Andi Alijagic, Angela Bonura, Francesco Barbero, Victor F. Puntes, Francesco Gervasi, Annalisa Pinsino

**Affiliations:** 1 Consiglio Nazionale delle Ricerche, Istituto per la Ricerca e l’Innovazione Biomedica (IRIB), 90146 Palermo, Italy; andialijagic@gmail.com (A.A.); angela.bonura@irib.cnr.it (A.B.); 2 Catalan Institute of Nanoscience and Nanotechnology (ICN2), CSIC and BIST, Campus UAB, Bellaterra, 08193 Barcelona, Spain; fra.barbero@gmail.com (F.B.); victor.puntes.icn@gmail.com (V.F.P.); 3 Institució Catalana de Recerca i Estudis Avançats (ICREA), 08010 Barcelona, Spain; 4 Vall d Hebron Institut de Recerca (VHIR), 08035 Barcelona, Spain; 5 Specialistic Oncology Laboratory Unit, ARNAS Hospitals Civico Di Cristina e Benfratelli, 90127 Palermo, Italy; francesco.gervasi@arnascivico.it

**Keywords:** proxy for human model, quiescence and immune activation, nanointeraction, PVP–AuNPs antimicrobial function, infected sea urchin immune cells

## Abstract

We investigated the role of the gold nanoparticles functionalized with polyvinylpyrrolidone (PVP–AuNPs) on the innate immune response against an acute infection caused by *Vibrio anguillarum* in an in vitro immunological nonmammalian next-generation model, the sea urchin *Paracentrotus lividus*. To profile the immunomodulatory function of PVP–AuNPs (0.1 μg mL^−1^) in sea urchin immune cells stimulated by *Vibrio* (10 μg mL^−1^) for 3 h, we focused on the baseline immunological state of the donor, and we analysed the topography, cellular metabolism, and expression of human cell surface antigens of the exposed cells, as well as the signalling leading the interaction between PVP–AuNPs and the *Vibrio*-stimulated cells. PVP–AuNPs are not able to silence the inflammatory signalling (TLR4/p38MAPK/NF-κB signalling) that involves the whole population of *P. lividus* immune cells exposed to *Vibrio*. However, our findings emphasise the ability of PVP–AuNPs to stimulate a subset of rare cells (defined here as Group 3) that express CD45 and CD14 antigens on their surface, which are known to be involved in immune cell maturation and macrophage activation in humans. Our evidence on how PVP–AuNPs may stimulate sea urchin immune cells represents an important starting point for planning new research work on the topic.

## 1. Introduction

Gold nanoparticles (AuNPs) play a key role in the development of nanoresearch (especially nanomedicine research), mainly due to the progress made in the synthesis and surface functionalization of particles, which has led to the development of next-generation materials with high biocompatibility [1]. More than 1000 methods are currently applied for synthesizing AuNPs, including chemical, physical, and biological synthesis [2]. The specificity, selectivity, and sensitivity of AuNPs for targeted applications in biomedicine (from diagnosis to therapy) are obtained by functionalizing the particles with a wide range of matters, including polymers, surfactants, drugs, nucleotides, metabolites, and peptides [3]. Functionalized AuNPs attract a large amount of attention, especially for their binding property with organic molecules (e.g., lipids, sugars, nucleic acids, and particularly proteins), their influence on the cytotoxicity and cellular uptake by cells, and their strong absorption spectrum [4,5]. For example, photothermal therapy using AuNPs is a promising tool in tumour therapy [6]. These particles are also promising drug carriers able to increase the antibacterial efficiency of antibiotics against some resistant bacteria [7]. In most cases, the interaction of nanoparticles with an organism results in a physiological immune response, which removes the possible harm with mechanisms that re-establish cellular homeostasis and tissue function [8]; this interaction can even improve the antioxidant capacity, immunity, and growth performance of an organism [9,10]. On the other hand, the nanoscale interaction with particles (e.g., nude particles) may lead to toxico–pathological consequences. In general, the concentration and size make a difference, and the same nanoparticles that fail to induce an adverse immune response at a low dose can trigger a pathological reaction at higher concentrations and smaller sizes [11].

Based on specific parameters of the AuNP design, it is possible to predict the biological interaction of particles with the immune system (the primary defensive barrier of an organism) to activate downstream immune signalling that may elicit both anti- and proinflammation, thus making it useful for a specific therapeutic plan [12]. Our previous works addressed the question based on the responsible use of animals in biomedical research (the 3R principle) [13], focusing on a new methodology for in vitro/ex vivo nanostudies based on a nonmammalian model that is a proxy for humans, the sea urchin (marine invertebrate) [14]. Several studies have displayed the suitability of the sea urchin as a model for risk assessment in environmental nanotoxicology [15,16]. Our recent report on sea urchin immune cells exposed to AuNPs functionalized with polyvinylpyrrolidone (PVP–AuNPs, 1 and 10 μg mL^−1^) underlined the capability of particles to activate a transient innate immune response independently of the concentration used [5]. PVP–AuNPs may be considered immunologically safe in this organism as they do not cause pathological inflammation, and findings could be translated to other organisms, including humans [17]. Polyvinylpyrrolidone (PVP) is a large biocompatible, chemically stable, and harmless polymer; it increases the solubility of drugs both in water and organic solvents and inhibits particle aggregation [18,19].

Bacterial infections are one of the major reasons for diseases and death, and the widespread use of antibiotics has led to the emergence of multidrug-resistant bacterial strains [20]. This public health problem involves not only humans but also plants and animals, with strong implications for farming (e.g., agriculture, livestock farming, and aquaculture).

Nanoparticles are becoming a promising tool to counter bacterial infections. For example, they are employed as antibacterial coatings on medical implants both to avoid infection and stimulate wound healing, as carriers for traditional antibiotics for targeting the pathogens intracellularly, and as tools for detecting the pathogens and as antibacterial vaccines [21]. However, the capability of nanoparticles to act as potential adjuvants able to modulate the function of innate immune cells to a bacterial infection remains scant.

The present work aims to investigate the possible role of the PVP–AuNPs in the innate immune response against an acute *Vibrio* (*Vibrio anguillarum*) infection in the in vitro immunological nonmammalian model of the next-generation, Mediterranean sea urchin, *Paracentrotus lividus*. *Vibrio anguillarum* causes haemorrhagic septicaemia (known as vibriosis), affecting several farmed fish and shellfish, including sea urchins. This disease is responsible for severe economic losses in the fishing and aquaculture industries worldwide [22]. Recent studies report increasing human illness associated with *Vibrio* in the United States [23], especially in immunocompromised individuals [24]. Bacterial identification can be performed directly from positive blood cultures using a high throughput screening [25]. To investigate the putative immunomodulatory effects of PVP–AuNPs (0.1 μg mL^−1^) in *Vibrio*-stimulated sea urchin immune cells (10 μg mL^−1^) from donors under immunologically active (short-term housed sea urchins) and quiescent (long-term housed sea urchins) states, here, we focus on: (i) PVP–AuNP behaviour in the sea urchin coelomic fluid (CF); (ii) particle–bacteria-immune cell interface (cell topography and cell viability/metabolic activity) and flow cytometric analysis of the cells based on the baseline immunological state of the donor; (iii) defensive innate/inflammatory signalling leading immune-PVP–AuNP-infected cell interaction in vitro. We demonstrate that PVP–AuNPs stimulate a small subpopulation of poorly represented cells, which is suggested to have a role in protecting cells against the pathogen attack independently of the immunological state of the donor, but they are not able to silence the inflammatory signalling that involves the total cell population against an excessive injury that leads to failure of resolution and even the cellular death.

## 2. Materials and Methods

### 2.1. Synthesis and Characterization of Gold Nanoparticles Coated with Polyvinylpyrrolidone

PVP–AuNPs were prepared as already reported by Alijagic et al. [5]. Briefly, PVP in milliQ water plus sodium citrate (Na_3_Ct)-coated AuNPs was stirred overnight, PVP–AuNPs were purified from the surplus of PVP, the supernatant was removed, and particles were resuspended in the water and washed twice. The final concentration of the PVP–AuNP was 2 μM PVP coated on 100 μg mL^−1^ AuNPs.

The particle microstructure was characterized by a scanning electron microscope (SEM; FEI Magellan 400 XHR; eXtreme High Resolution). PVP–AuNPs (4 μL) were dispersed onto a carbon-coated copper transmission electron microscopy (TEM) grid and dehydrated at room temperature. PVP–AuNPs were analysed for their behaviour in the Coelomocyte Culture Medium (CCM) (Henson et al. [26] slightly modified by Pinsino and Alijagic [27]) and in the sea urchin cell-free coelomic fluid plus CCM (UV–vis spectroscopy analyses), as already described [5]. Due to the detection limits of the methods employed for the particle characterization, particles were first characterized at the highest concentrations (0.2, 1 and 10 μg mL^−1^), and the results were used to elaborate and predict the behaviour of particles at the concentration of 0.1 μg mL^−1^.

### 2.2. Preparation of Bacteria

*Vibrio anguillarum* (*Vibrio*, Gram-negative) was purchased from the Carolina Biological Supply Company (Burlington, NC, USA). Cultures were grown for 3–4 days at 25 °C in a sterilized 4% Lysogeny broth (LB) medium (Sigma, USA) by rotating shaker incubation. The colony-forming units (CFU) were determined by Biophotometer D30 (Eppendorf, Hamburg, Germany), at the optical density of 600 nm (OD600 = 1 = 10^8^ CFU/mL). Cultures were collected, centrifuged, and washed three times at 4500× *g* for 15 min at 4 °C. The CFU was remeasured, and bacteria were resuspended in artificial seawater. Bacteria were heat-killed at 95 °C, divided into aliquots, stored at −20 °C, and used (after defrosting) as an immunological challenge, as described below.

### 2.3. Sea Urchin, Immune Cell Exposure, and Cell Viability Assay

Adult sea urchins (*Paracentrotus lividus*) were collected from the coasts of Sicily (Italy), gradually acclimatized, and kept under strictly controlled conditions [27]. To study immunological responses induced by *Vibrio* and PVP-coated AuNPs, we used housed donors from 2–4 weeks (short-term housed sea urchins) to 6–8 months (long-term housed sea urchins). Immune cells were harvested from each donor through the soft peristomal membrane by sterile 27-gauge needles containing sterile ice-cold anticoagulant solution CCM (1:1 ratio) [27] and exposed to the PVP–AuNPs (0.1 μg mL^−1^), the *Vibrio* (10^6^ CFU/mL or 10 μg mL^−1^), and the particle–bacteria combination (0.1 μg mL^−1^ PVP–AuNPs *plus* 10 μg mL^−1^ *Vibrio*). The exposures were performed in tubes of 15 mL placed on a shaking platform for 3 h at 16 ± 2 °C (dynamic culture). The time of exposure to *Vibrio* was selected to activate an acute inflammation for studying the influence of the particle on the immune cell function under a state of altered responsiveness (e.g., sepsis). After exposure, immune cells were collected via centrifugation (9000× *g*) and stored at −80 °C for biochemical analyses. For imaging, 1.5 × 10^6^ immune cells were seeded in a 24-well plate (Thermo Fisher Scientific, East Grinstead, UK) in a static culturing system, as recently described [27]. Micrographs of immune cells were acquired using an inverted microscope (OLYMPUS CKX31, Olympus, Tokyo, Japan).

Immune cell viability was evaluated in real time using the nonlytic and bioluminescent RealTime-Glo MT Cell Viability Assay (Promega, Madison, WI, USA) by measuring the reducing potential of cells as previously described [27], with slight modifications. Immune cells were incubated for 5 min with the particle, bacteria, and related combination under dynamic conditions. Later, 1 × 10^5^ cells per treatment were transferred in the 96-well microplate to a final volume of 100 μL. The assay involved seventeen biological replicates (immune cell samples from 17 donors). Measurements were expressed both as relative luminescence units (RLU) and arbitrary units.

### 2.4. Flow Cytometry

Immune cells exposed to the PVP–AuNPs, *V. anguillarum*, and particle–bacteria combination were analysed after an incubation period of ~4 h at 16 ± 2 °C in tubes placed on a shaking platform under gentle agitation (dynamic culture) as reported above. Immune cell populations were defined via flow cytometry through the analysis of forwarding scatter/side scatter (FS/SS) density plots based on their intrinsic size (FS) and granularity (SS) using a logarithmic scale (Flowjo 10.7.1, LLC). Cells were incubated with one of the following antihuman monoclonal antibodies targeting human cell surface antigens in vivo: CD45-FITC (Beckman Coulter, Krefeld, Germany, A07782) and CD14-PC5.5 (Beckman Coulter, A70204). Monoclonal antibodies were added in amounts of 300–500 μL of cell suspension and incubated for 20 min at 4 °C. After incubation with antibodies, the cell suspension was immediately analysed in a Navios EX flow cytometer (Beckman Coulter). A minimum of 10,000 cells per sample were examined, and the collected data were analysed using the Kaluza analysis software. The assay involved four biological replicates (immune cell samples from four donors). The pulse height versus pulse width plots were used to isolate single cells passing through the cytometer, thereby excluding any nonsingle cells (e.g., doublets, clumps, and entrapped debris). The levels of autofluorescence were determined using unstained cells (both control and exposed cells) collected with and without the anticoagulant solution. As there is less autofluorescence at longer light wavelengths, fluorophores that emit above 500 nm were selected (e.g., FITC, 519 nm; PE-Cy5.5, 700 nm) to limit the autofluorescence interference.

### 2.5. SDS-PAGE and Immunoblotting

Total protein content was measured using the BioRad assay kit (Hercules, CA, USA); 15 µg of proteins per sample were mixed with acetone (1:1) and precipitated overnight at −20 °C. Protein pellets were suspended in SDS sample buffer, denaturized, separated on 4–20% Mini-PROTEAN Tris-Glycine eXtended (TGX) precast polyacrylamide gels (BioRad, Berkeley, CA, USA), and transferred to a nitrocellulose membrane (Amersham, UK) as previously described by Alijagic et al. [5]. Following blocking, membranes were incubated with the following primary antibodies: (i) anti-*Pl*-toposome (BEVIB12b8) 1:200 [28]; anti-*Pl*-nectin 1:200 [29]; anti-*Pl*-galectin-8 1:800 [30]; anti-TLR4 (H-80) (Santa Cruz Biotechnology, sc-10741) 1:250; anti-HSP70 (SIGMA, Burlington, MA, USA, Cat N. H-5147) 1:1000; anti-phospho-p38 MAP Kinase (Tr180/Tyr182) (Cell Signalling, Danvers, MA, USA, 9211) 1:250; Phospho-p42/44 MAP Kinase (ERK1/2) (Cell Signalling, 9101) 1:300; IL-6 (H-183) (Santa Cruz Biotechnology, Dallas, TX, USA, sc-7920) 1:100; anti-MnSOD (Enzo Life Sciences, ADI-SOD-111) 1:200; anti-NF-κB p65 (H-286): sc-7151 1:200; anti-β-actin (SIGMA, A5441) 1:500. Following rinsing, membranes were incubated with a fluorescein-labelled secondary antibody (LI-COR Biosciences, Lincoln, NE, USA) and visualized using the Odyssey Infrared Imaging System (LI-COR Biosciences). The assay involved six biological replicates (immune cell samples from six donors).

### 2.6. Statistics

Statistical analyses were performed using the GraphPad Prism Software 6.01 (USA). The statistical significance was set to *p* < 0.05. Data were shown as mean ± standard deviation (SD).

## 3. Results and Discussion

### 3.1. Polyvinylpyrrolidone Leads to the Formation of Hetero-Aggregates Independently of the Concentration and Time Exposure of the Gold Particles in the Sea Urchin Coelomic Fluid

Nanoparticles exposed to different biological and environmental scenarios tend to reach a more stable thermodynamic state through homoaggregation (particle–particle) and/or interaction with the biomolecules and other organic matter present in the environment (heteroaggregation) [31]. These transformations change the nature of the nanobjects, their behaviour, and their potential risks. The transformation can occur even if nanoparticles are functionalized by coating molecules (including PVP) [32]. Thus, the same nanoparticles can have a different impact on biological systems and the environment depending on the microenvironment (e.g., the exposure medium) in which the particles are dispersed [33].

The coelomocyte culture medium (CCM) is a high-salt medium that presents a density and salinity close to the sea urchin coelomic fluid, preserves the morphological structures of sea urchin immune cells, and blocks calcium-dependent cell coagulation [27]. Thus, the CCM is necessary for performing sea urchin primary immune cell cultures, as recently reported by Pinsino and Alijagic [27].

**Figure 1:**
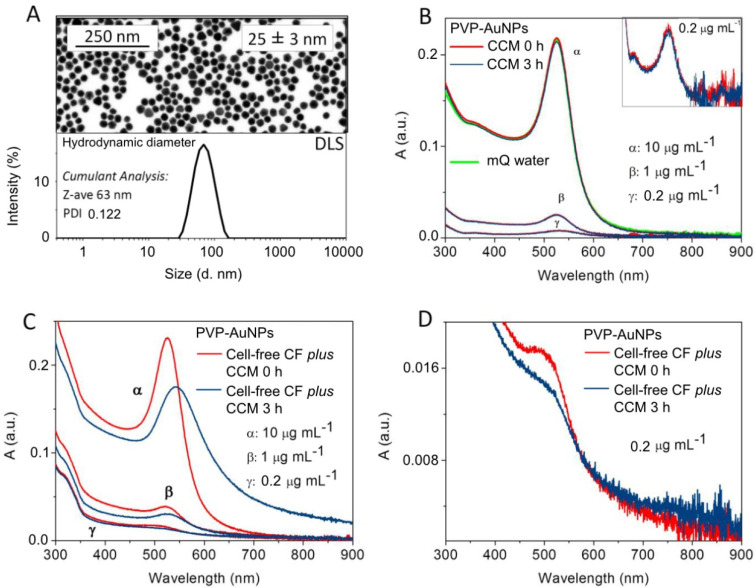
PVP–AuNP behaviour in the sea urchin culture media. (**A**) SEM image of PVP–AuNPs (upper panel); DLS size distribution of PVP–AuNPs (10 µg mL^−1^) dispersed in CCM at 3 h of exposure (lower panel). (**B**) UV-vis spectra of PVP–AuNPs (0.2, 1, 10 μg mL^−1^) dispersed in CCM at 0 (red line) and 3 h (blue line) of exposure, and in mQ water (green line). (**C**) PVP–AuNPs dispersed in cell-free coelomic fluid (CF) plus CCM at 0.2, 1, and 10 μg mL^−1^ and 0 and 3 h of exposure (red line and blue line, respectively). (**D**) PVP–AuNPs dispersed in CF cell-free plus CCM at 0.2 μg mL^−1^ and 0 and 3 h of exposure (red line and blue line, respectively).

Nanoparticles exposed to high-salt mediums tend to aggregate/agglomerate, acquiring a much larger size, which changes their optical properties. The PVP features influence nanoparticle growth and morphology by maintaining particle dispersity in different mediums and supplying selective surface stabilization and kinetically controlled growth conditions [34]. PVP–AuNPs (1 and 10 μg mL^−1^) present good monodispersity and colloidal stability after 24-h dispersion in CCM, as recently described [5]. Here, we extended our previous study focusing on the physicochemical characterisation of PVP–AuNPs dispersed in CCM and cell-free coelomic fluid plus CCM at a shorter time of dispersion/exposure (from 0 to 3 h), and lower concentration (0.1 μg mL^−1^). The PVP–AuNPs (core diameter of 25 ± 3 nm) showed a hydrodynamic diameter of 63 nm with a polydispersity index (PDI) of 0.122 at 3 h of dispersion in CCM (Figure 1A), and a zeta potential value of—4.1 ± 0.6 mV (conductivity 29.1 mS cm^−1^). The UV–vis spectrum of PVP-AuNPs (0.2, 1 and 10 μg mL^−1^) dispersed in CCM for 3 h presented a surface plasmon resonance band peaking at 524 nm, consistent with a monodisperse particle profile (Figure 1B), in agreement with the results obtained at 24 h of exposure [5]. The UV–vis spectrum of PVP-AuNPs (0.2, 1 and 10 μg mL^−1^) dispersed in cell-free coelomic fluid plus CCM showed a few substantial changes: (i) a broader and shifted plasmonic peak; (ii) a decrease in the peak absorption value; and (iii) an increase in the baseline value (Figure 1C,D), in line with events of surface changes [35,36]. Notably, on the left side of the plasmonic peak (550 nm), the red line decreased in comparison to the blue line, while on the right side of the plasmonic peak, the blue line increased due to particle surface modification and aggregation.

Results showed that the sea urchin extracellular biomacromolecules probably replace the PVP from the particle surface already after 3 h of exposure, thus leading to the formation of heteroaggregates. Sea urchin coelomic fluid is a major source of bioactive molecules, which are secreted by immune cells to maintain homeostasis and intercellular crosstalk within the organism [37]. PVP–AuNPs interact with sea urchin extracellular bioactive molecules forming a biocorona based on a few proteins within 24 h, as already described [5]. As the behaviour of PVP–AuNPs is independent of the particle concentration (within the tested orders of magnitude), we can assume that at 0.1 μg mL^−1^ the particles dispersed in the sea urchin coelomic fluid also undergo the same conditions as observed above.

### 3.2. The In Vitro Particle–Bacteria–Immune Cell Interface: How Cells Behave Differently Based on the Baseline Immunological State of the Donor

A heterogeneous free-swimming population of cells present in the fluid of the coelomic cavity mediates immunity in adult sea urchins [38]. Three major subsets of immune cells have been described in the Mediterranean sea urchin, *P. lividus* (phagocytes, red and white amoebocytes, and vibratile cells) with functions similar to those of human immune cells, including self/non-self-recognition, phagocytosis, rejection, cytotoxic activity and destruction of bacteria and others harmful hosts or chemicals [39]. Additionally, they can initiate inflammation by releasing functional biomolecules and inducing an immune signalling cascade [40,41].

**Figure 2:**
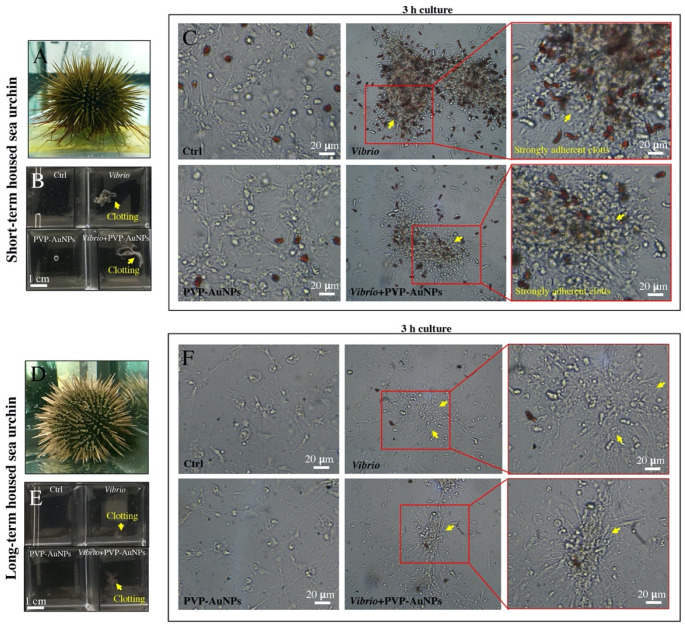
Sea urchin immune cells under in vitro exposure. (**A**) Short-term housed *P. lividus* (2–4 weeks). (**B**) Macroscopic overview of the immune cells from short-term housed sea urchins after a few seconds of exposure (*V. anguillarum*, PVP–AuNPs, *Vibrio plus* PVP–AuNPs). (**C**) Sea urchin immune cells from short-term housed sea urchin after 3 h of exposure in vitro. (**D**) Long-term housed *P. lividus* (6–8 months). (**E**) Macroscopic overview of the immune cells from long-term housed sea urchins after a few seconds of exposure (*V. anguillarum*, PVP–AuNPs, *Vibrio* *plus* PVP–AuNPs). (**F**) Sea urchin immune cells from long-term housed sea urchin after 3 h of exposure in vitro.

In the natural sea environment, the sea urchin immune system is continuously stimulated by pathogens; thus, the baseline immunological state in freshly caught animals is more active than that of animals kept in tanks for a long time [42]. Here, an acute infection was induced by exposing cells from animals kept in tanks for 2–4 weeks (short-term housed sea urchins) and 6–8 months (long-term housed sea urchins) to high concentrations of the heat-killed marine bacterium *Vibrio anguillarum* (10 μg mL^−1^) for 3 h (Figure 2). Although there are several morphologically distinct classes of *P. lividus* immune cells, the phagocytes are the most abundant subset and appear to play a major role in the immune response against pathogens [38]. Under *Vibrio* exposures, the phagocytic cells aggregated immediately (Figure 2, see yellow arrows), activating the cellular encapsulation of the pathogen (clotting formation). Clotting acted as the first defence mechanism function against *Vibrio*, whereas it was not activated against nanoparticles. The other types of cells were passively trapped in the surface of clots (Figure 2C,F). Notably, clotting formation in immune cells from sea urchins housed in tanks for a short time was stronger than that in immune cells from animals kept in tanks for a long time (compare Figure 2C,F), highlighting the fact that cells from donors housed for a short time were much more reactive. Moreover, the immune cells from sea urchins kept in tanks for a long time showed a decrease in the total number of the cells freely circulating in the coelomic fluid (1.3 ± 0.6 × 10^6^ cells/mL of CF plus CCM, mean value ± SD of seven samples) compared to those from a short time (4.6 ± 0.8 × 10^6^ cells/mL of CF, mean value ± SD of nine samples), including a reduction in the red amoebocyte content (compare Figure 2C,F). This is in agreement with the notion that long-term housing induces sea urchin immunological quiescence, but not immunological suppression, as the immune activation can be reversed with injections of bacteria or other harmful hosts or chemicals, a few days after the first of three repetitive exposures [42]. Notably, the topography of immune cells exposed to PVP–AuNPs (0.1 μg mL^−1^) was not different from that of the control cells. Cells were securely attached to the culture plate and organised in a network of bundles and fibres, regardless of the time of housing of the donor (Figure 2C,F, left panel). Particles did not affect the cellular behaviour and interfered only slightly with the clotting formation induced by *Vibrio* infection (compare Figure 2C,F, the middle panel).

**Figure 3:**
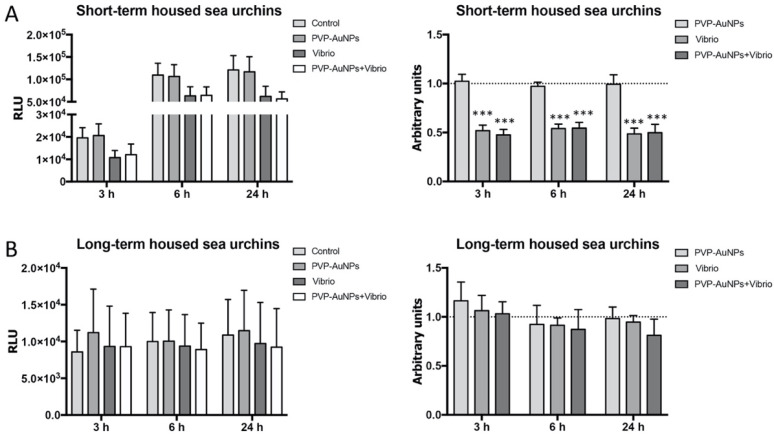
Real-time viability assay reveals how the baseline immunological state interferes with the capability of the cells to sense the infection. The cellular viability/metabolism of the immune cells exposed to PVP–AuNPs (0.1 μg mL^−1^), *Vibrio anguillarum* (10 μg mL^−1^), and particle–bacteria combination was measured in cells from both short-term housed donors (**A**) and long-term housed donors (**B**). The number of donors was 12 short-term and 5 long-term housed sea urchins. Levels are expressed in both relative luminescence unit (RLU) (on the left), and arbitrary units (fold increase or decrease compared to controls set to 1) (dotted line) (on the right). Data are reported as the mean ± SD; asterisks (*) indicate significant differences among groups (*** *p* < 0.001).

The RealTime-Glo MT Cell Viability Assay provided information on the metabolic activity of cells (Figure 3). Measurements expressed as RLU indicated an increasing trend in the metabolic activity of the cells from short-term housed donors compared to those from long-term housed donors (compare Figure 3A,B, left panel). Concordantly, in some systems (e.g., *Saccharomyces cerevisiae*, mammalian lymphocytes and hematopoietic stem cells), cells in quiescence show a low metabolism expressed by a decrease in glucose uptake, and an increase in autophagy as a useful survival strategy [43]. Metabolites are guarded by extracellular signals, and quiescence signals often inactivate the mammalian target of the rapamycin (mTOR) signalling pathway, resulting in reduced cell growth and protein synthesis. In turn, metabolites regulate the immunological reactivity (e.g., duration and intensity) and innate memory [44].

Measurements expressed as arbitrary units showed that the viability of the cells from short-term housed donors incubated with PVP–AuNPs (0.1 μg mL^−1^) for 24 h was not different from the controls (cells cultured in the absence of particles) (Figure 3A, right panel). On the contrary, the viability was significantly decreased for cells incubated with the *Vibrio* and the particle–bacteria combination compared to controls (Figure 3A, right panel), probably due to clotting. Clotting undergoes a rapid process of fusion and cell death forming a cellular mass in a process strongly analogous to thrombosis [45]. This is the major cause of host elimination following inflammasome activation, and phagocytosis occurs in the clot neighbourhood [46]. Sea urchin immune cells express a large number of scavenger receptor cysteine-rich (SRCR) genes, part of which are cell–cell adhesion molecules in this organism [47,48], and, as in human macrophages, have a role in clot formation [49]. The viability of the cells from long-term housed donors incubated with particles, bacteria, and the related combination did not display a significant difference compared to control cells (Figure 3B, right panel); only a slight reduction in the viability of all cells (both exposed and unexposed) was found (Figure 3B, left panel), in line with the reduced ability of these cells to be promptly activated due to quiescence.

### 3.3. Polyvinylpyrrolidone-Functionalized Gold Nanoparticles Try to Interfere with the Immunological State Affected by Vibrio Anguillarum

**Figure 4:**
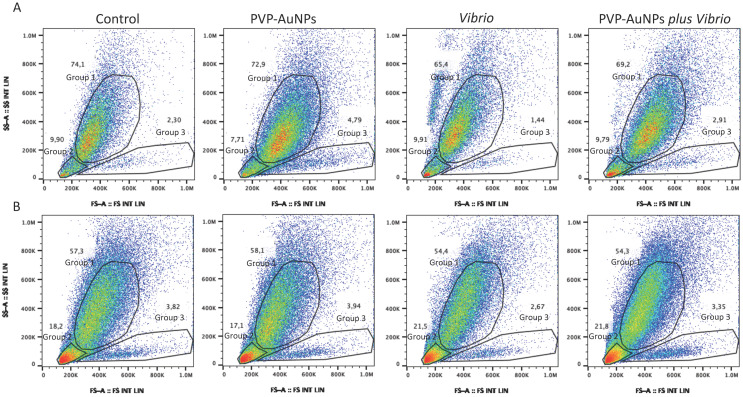
Two-parameter flow cytometric analysis of immune cells of an immunologically quiescent sea urchin *Paracentrotus lividus* via forward scatter (FSC) and sideward scatter (SSC). (**A**) Forward/side scatter profile of total immune cells harvested from the donor in the coelomic calcium medium (CCM, anticoagulant solution): unexposed cells (control), and cells exposed to PVP–AuNPs (0.1 μg mL^−1^), *Vibrio* (10 μg mL^−1^), and particle–bacteria combination. (**B**) Forward/side scatter profile of total immune cells harvested from the same donor without CCM. The gating of distinct populations is shown, and the percentage of cells in each of these gates is provided.

Flow cytometry represents a powerful tool that is widely used to identify and characterize cells within complex populations. Here, this tool was used to identify the presence and proportion of those subpopulations of the *P. lividus* immune cells involved in the immune response against nanoparticles and bacteria. Two-parameter flow cytometric separation of the cells by forward scatter (FS) and sideward scatter (SS) allowed us to identify two major groups of cells that we assumed to be phagocytes (Group 1, upper gate), exhibiting a high degree of internal complexity (SS) and size (FS), and amoebocytes and vibratile cells (Group 2, lower left gate), exhibiting a small degree of complexity and size; as well as one small group of poorly represented large cells with a small degree of complexity (Group 3, lower right gate) (Figure 4, representative image of one of the four donors).

The phagocyte population exposed to PVP–AuNPs (0.1 μg mL^−1^, 4-h exposure) underlined a shift in the FS signal intensity from the left to the right (From 400 to 600k) compared to the control, indicative of the increased size of the cells, probably due to the fact that they have taken up particles (Figure 4A), in agreement with Romero et al. [50]. On the contrary, cells exposed to *Vibrio* (10 μg mL^−1^, 4-h exposure) showed a consistent shift to the left (below 200 k) with an increase in complexity, probably due to the clotting formation, whereas cells exposed to the particle–bacteria combination showed both features described above (Figure 4A). The dot plots from cells harvested and exposed without an anticoagulant solution confirmed that the increased complexity of the exposed cells is consistent with both the clotting and phagocytosis (Figure 4B). The image also identified debris consistent with the cell–cell fusion and cell death leading to clot formation (Figure 4B, Group 2; see the large red spots). Notably, the small subpopulation of poorly represented cells (large in size and small in complexity) was found to be slightly decreased in response to *Vibrio* compared to controls (Figure 4A, Group 3; the results of one of the four donors are shown), independent of the baseline immunological state of the donor (immunologically active or quiescent). Conversely, exposure to PVP–AuNPs stimulated a slight or no increase in this subpopulation. In immunologically quiescent *P. lividus* donors, particles helped cells exposed to *Vibrio* (particle–bacteria combination) maintain the number of cells falling within Group 3 close to the control, much more than in immunologically active donors (Figure 4A and Table S1 reported in Supplementary Materials).

Smith et al. [51] subcategorized the sea urchin phagocytes into three morphotypes: discoidal phagocytes, polygonal cells, and small phagocytes. We speculate that the subsets of cells that defined Group 3, may reflect an intermediate state of maturity (precursors) before becoming mature phagocytes [52]. Concordantly, cells of zebrafish evaluated by flow cytometry exhibited three distinct cell populations according to their size and granularity (complexity): (i) macrophage/monocytes and granulocytes; (ii) hematopoietic precursors; and (iii) lymphocytes and lymphocyte-like cells [53]. The exact origin of sea urchin immune cells is still a matter of debate. The coelomic epithelium is the preferential hematopoietic tissue, as proposed by the pioneering work of Bossche and Jangoux [54] and further supported by more recent studies [55,56]. Thus, the freely circulating sea urchin immune cells probably do not divide but are derived from progenitor cells that complete cell maturation into the coelomic fluid [57].

The exploration of the sea urchin genome has revealed strong similarities between sea urchin and human innate immune-related genes [48]. Based on the notion that the sea urchin immune system activates mechanisms that are strictly related to those occurring in humans [17], we assayed the binding of sea urchin immune cells (falling within Group 3) stimulated by PVP–AuNPs to human antigens by flow cytometry. A few fluorescently labelled antibodies targeting human cell surface antigens have been used for this purpose, including CD45-FITC and CD14-PE-Cy5.5. It is well known that larger cells and more granular cells show high levels of autofluorescence due to the high number of fluorescent complexes. The autofluorescence was minimal for both light wavelengths used as expected (not shown).

CD45 is a receptor-like protein tyrosine phosphatase expressed on hematopoietic cells, and plays a central role in several cellular processes, including proliferation, maturation, and antiviral responses of the cells [58]. Additionally, CD45 can affect immune cell adhesion and modulate intracellular signalling involved in the immune responses [59].

The number of CD45-positive cells was decreased upon exposure to *Vibrio* in both immunologically quiescent donors, compared to controls (−5.5 ± 4.6 mean ± SD) (see Table 1). Upon exposure to *Vibrio* in combination with particles, cells from immunologically quiescent donors showed a percentage of CD45-positive cells higher than those of cells exposed to *Vibrio* alone (+2.2 ± 0.3 mean ± SD). In immunologically active donors, the above scenario is not so well defined. In one sample of two, the number of CD45-positive cells was increased upon exposure to *Vibrio* compared to controls, while in two samples of two the number of CD45-positive cells was increased in cells exposed to *Vibrio plus* PVP–AuNPs compared to those exposed to *Vibrio* alone. The number of CD45-positive cells was increased in cells exposed to PVP–AuNPs in just one immunologically quiescent donor and both immunologically active donors. CD45 is known to be a regulator of Toll-like receptor (TLR)-mediated cytokine secretion in mammalian dendritic cells; it is not required for the development of these cells but influences cell maturation [60]. Notably, when CD45 binds to Toll-like receptor 4 (TLR4), the content of the proinflammatory cytokines decrease, suggesting that CD45 regulates negatively the TLR4 signalling pathways [60]. We speculate that the increased number of CD45-positive cells stimulated by the PVP-AuNPs may be related to an increased number of cells that become differentiated.

The same trend was observed for CD14, with the only exception being the number of CD45-positive cells upon exposure to PVP–AuNPs (increase in just one immunologically active donor and two quiescent donors compared to controls).

In summary, the immune system of quiescent donors behaved in a stable way, showing a low metabolism, a decrease in the number of cells falling within Group 3, and a decrease in the number of cells positive for CD45 and CD14 when affected by *Vibrio.* On the contrary, the immune system of active donors behaved more variably based on the individual basic capability to react with greater or lesser reactivity to the infection. Thus, the known variability in immunological parameters among individuals did not permit us to highlight of statistically significant differences, but in all cases, the number of CD14- and CD45-positive cells was increased in immunologically active or quiescent samples exposed to particles in combination with bacteria (PVP–AuNPs *plus Vibrio)* compared to *Vibrio*-exposed samples (see Table S2 reported in Supplementary Materials). PVP–AuNPs increase the number of this cell type acting as an anti-inflammatory agent in immunologically active or quiescent cells exposed to *Vibrio*.

CD14 is a lipopolysaccharide-binding protein expressed on human monocytes and macrophages. TLR4 is a CD14 signalling coreceptor, which triggers an inflammatory signalling cascade dependent on the nuclear factor κB (NF-κB) [61]. Human monocytes expressing CD16 with reduced CD14 (CD14^+^CD16^+^ monocytes) cause inflammatory diseases, sepsis and bacteraemia [62]. Additionally, CD45 also plays a role in lipopolysaccharide (LPS)-induced responses in macrophages through the CD14 pathway, as proven by Pfau et al. [63]. Based on our findings, we speculate that the sea urchin immune cell subpopulation, here known as Group 3, is a subset of cells involved in several aspects of macrophage-like activation depending on the TLR4/NF-κB signalling pathway (e.g., maturation and inflammation).

**Figure 5:**
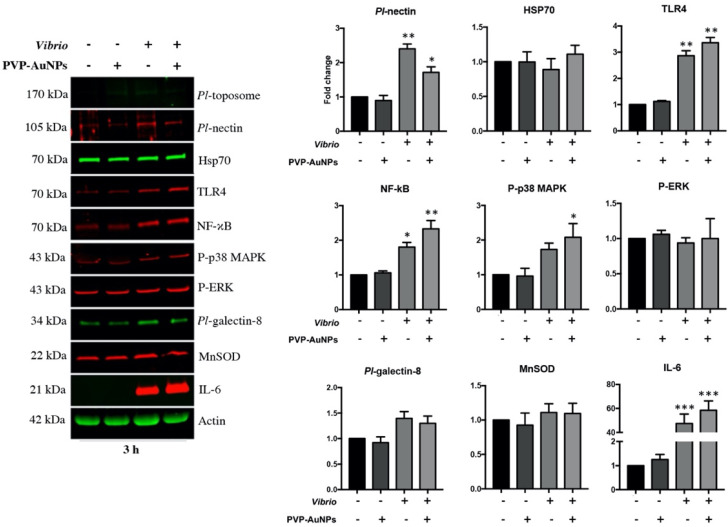
PVP–AuNPs are not able to block the powerful inflammatory response involving the TLR4/NF-κB signalling pathway activated by *Vibrio*. Representative immunoblotting shows results for the selected subset of proteins in cells exposed to PVP–AuNPs (0.1 μg mL^−1^), *Vibrio* (10 μg mL^−1^) and particles in combination with bacteria for 3 h. Histograms are representative of the means ± SD of six replicates after normalisation with actin. Protein levels are reported in arbitrary units (fold increase or decrease compared to controls that are set to 1). Here, a representative image of the protein levels obtained from three individual donors per group is reported. * *p* < 0.05, ** *p* < 0.01, *** *p* < 0.001.

To support our speculation, we outlined the immunological state of the whole population of *P. lividus* immune cells exposed to PVP–AuNPs, *Vibrio*, and particles in combination with bacteria, based on changes in the levels of a few proteins involved in the defensive response in cells from immunologically active donors. Notably, we focused on the detection of proteins triggering immune and stress response (TLR4, NF-κB, Heat shock protein 70, Interleukin-6, Manganese superoxide dismutase, 38 mitogen-activated protein kinase, and p42/44 MAP Kinase), and on those cellular adhesion proteins that are well-characterized in the *P. lividus* (*Pl*-toposome, *Pl*-nectin, and *Pl*-galectin-8) (Figure 5). The results of the *P. lividus* immune cells exposed to PVP–AuNPs (0.1 μg mL^−1^, 3 h-exposure) were consistent with our prior findings on the general physiology of the exposed cells (Figure 2); their protein levels were very similar to those of the controls, confirming that particles did not activate an immunological response involving the TLR4/NF-κB signalling pathway, in agreement with our previous report on the immunogenicity of PVP–AuNPs (1 μg mL^−1^) in the sea urchin phagocytes exposed for 24 h in vitro [5]. Additionally, this finding was consistent with our observation on the increased number of CD45-positive cells stimulated by the PVP–AuNPs (see Table 1), as on the notion that CD45 may act as a negative regulator of the TLR4 signalling cascade by inhibiting the inflammatory response [60]. *Vibrio* is a common aquatic pathogenic bacterium in several organisms, including adult sea urchins and humans [64,65]. Sea urchin immune cells exposed to high concentrations of the heat-killed marine bacterium *Vibrio anguillarum* (10 μg mL^−1^, 3 h-exposure) activated a fast and pathologically inflammatory response involving the TLR4/p38 MAPK/NF-κB signalling pathway based on increased protein levels, culminating in the production and accumulation of the pro-inflammatory protein interleukin-6 (IL-6). This biochemical pathway, including the involvement of the CD14 that decreased in cells of the Group 3 exposed to *Vibrio* (see Table 1), emphasises that exposure to this pathogen (10 μg mL^−1^) drives inflammatory signalling against an excessive injury that leads to failure of resolution and even cellular death, in agreement with our findings on cell viability (Figure 3). Although PVP–AuNPs showed the ability to operate as a protective agent against *Vibrio* infection based on the timid signs of recovery promoted by the increase in the number of CD45- and CD14- positive cells of Group 3 (see Table 1: compare cells exposed to particles in combination with bacteria to cells exposed to *Vibrio*), they failed to promote the resolution of the innate inflammatory response (Figure 5, *Vibrio* plus PVP–AuNPs). In agreement, the TLR4, NF-κB and IL-6 protein levels of the cells exposed to particles in combination with bacteria for 3 h remain highly close to those of cells exposed to *Vibrio* alone. Exposure to *Vibrio* did not inhibit or enhance the level of the heat shock protein 70 (Hsp70), and the mitochondrial antioxidant enzyme, namely manganese superoxide dismutase (MnSOD). In agreement, no evident differences were found in the phosphorylation of p42/44 MAP Kinase (pERK), whereas the *Vibrio* exposure led to a statistically significant increase in the *Pl*-nectin cell adhesion protein levels, which is known to mediate the immune recognition of a pathogen in humans [66]. Stimulation of the TLR4/myeloid differentiation factor (MD2) complex by LPS triggers phosphoinositide 3-kinase (PI3K)/protein kinase B (AKT)/mTOR signalling and promotes b1 integrin, thereby increasing cellular adhesion [67]. The increased levels of the adhesion proteins are in agreement with the massive cellular clot/aggregation of the sea urchin immune cells exposed to *Vibrio* (Figure 2). Critically, we note that the biochemical results were obtained from the total cell population of the sea urchin immune cells exposed to particles, bacteria, and their combination, and not from the selected subpopulation to which we directed our attention above. It remains a challenge for future research to refine the experimental setup and conditions.

## 4. Conclusions

PVP–AuNPs do not harm the physiological health of sea urchin immune cells, preserving their immunological state. By investigating the immunomodulatory function of PVP–AuNPs in protecting against *Vibrio* infection in vitro we demonstrate that these particles stimulate a subset of rare cells, defined here as Group 3, which express two human cell-surface antigens on their surface, known to be involved in immune cell maturation and macrophage activation dependent on the TLR4/NF-κB signalling pathway. Results of the capability of these inert gold particles to promote mechanisms of action on the sea urchin immune cells against *Vibrio* infections are promising. Several reports have suggested that NPs (e.g., silver and gold) are toxic against a broad range of pathogens, especially in plants, fungi, viruses, and bacteria [68]. Exposure to high concentrations of the *Vibrio* activates a pathological inflammatory response with the impossibility of promoting a complete positive resolution. Further studies with a similar approach need to be performed in the future with a revised experimental setup (e.g., a lower concentration of *Vibrio*, a higher concentration of particles, and flow cytometry combined with single-cell sorting), to fully confirm the protective role of these nanoproducts against infections. Bacterial infections are a major cause of chronic infections and mortality. The widespread use of antibiotics has led to the emergence of multidrug-resistant bacterial strains and the development of new tools to contrast infection. Moreover, chronic infections correlated to aquatic bacteria are problematic to resolve due to their resistance to both host immunological responses and synthetic antibiotics [21]. Thus, finding alternatives to antibiotics is one of the greatest challenges facing the community of immunologists. The main antibiotic resistance mechanisms are irrelevant for nanoparticles as the mechanisms of action of the particles are directed against the bacterial surface. Studying the features of the immune responses from the use of gold nanoparticles against infections could permit the refinement of the use of particles as nanocarriers or adjuvants to produce antibiotics of next-generation, new vaccines against viral, bacterial and parasitic infections [69].

Our findings reinforce the notion that immune studies in emerging nonmammalian models should be implemented solving the apparent problems to successfully translate results to human immunity, in respect of the 3R principle of reducing, refining, and replacing animal experimentation. On the other hand, the control of microbial infection is essential to the successful farming of sea urchins which, together with molluscs, crustaceans, and fish, forms the basis of aquaculture production.

## Figures and Tables

**Table 1:** Functional characterization of the *P. lividus* immune cells, namely Group 3. The table reports percentage of the cells in the total cell population shown in Table S1, and the percentage of the cells stained with Anti-CD45-FITC and CD14-PE-Cy5.5 within the gate after doublet exclusion (to ensure to count single cells and exclude doublets from the analysis). CD45^+^: cells positive to Anti-CD45-FITC; CD14^+^: cells positive to CD14-PE-Cy5.5. Blue: *Vibrio* lower than control; red: PVP–AuNPs plus *Vibrio* higher than *Vibrio*; purple: *Vibrio* higher than control; yellow: PVP-AuNPs lower than control.

*P. lividus* Donor	Exposure Scenario	% of Cells after Doublet Exclusion	CD45^+^ (%)	CD14^+^ (%)
**Quiescent 1**	Control	**2.09**	17.83	20.43
	PVP-AuNPs	**3.33**	14.53	32.03
	*Vibrio*	**1.54**	2.06	6.41
	PVP-AuNPs *plus Vibrio*	**2.13**	4.02	12.47
**Quiescent 2**	Control	**11.84**	1.08	2.43
	PVP-AuNPs	**11.68**	1.54	3.28
	*Vibrio*	**9.47**	0.48	1.36
	PVP-AuNPs *plus Vibrio*	**12.12**	1.16	3.23
**Immunologically active 1**	Control	**9.66**	0.06	0.03
	PVP-AuNPs	**9.36**	0.16	0.05
	*Vibrio*	**8.82**	1.31	0.16
	PVP-AuNPs *plus Vibrio*	**8.60**	1.95	0.32
**Immunologically active 2**	Control	**10.10**	2.44	1.15
	PVP-AuNPs	**11.70**	4.25	0.43
	*Vibrio*	**7.73**	0.28	0.13
	PVP-AuNPs *plus Vibrio*	**7.35**	0.73	0.18

## Data Availability

The authors declare that the data supporting the findings of this study are available within the paper. All the other data are available from the corresponding author upon request.

## Supplementary Materials

The following are available online at https://www.mdpi.com/article/10.3390/nano11102646/s1, Table S1: Results from two-parameter flow cytometric analysis of immune cells of four sea urchin *Paracentrotus lividus*, Table S2: Individual values and the means ± standard deviations of the percentage of cells stained with Anti-CD45 and -CD14 from grouped quiescent and active *P. lividus* donors.
