# Supplementary material for: Immunomodulatory Function of Polyvinylpyrrolidone (PVP)-Functionalized Gold Nanoparticles in Vibrio-Stimulated Sea Urchin Immune Cells
Source: Nanomaterials (Basel). 2021 Oct 8;11(10):2646. doi: 10.3390/nano11102646 (PMC8539316; doi:10.3390/nano11102646)
Supplement: Supplementary file 1 [file nanomaterials-11-02646-s001.zip › nanomaterials-1334880-supplementary.pdf]

nanomaterials

Supplementary Materials

Immunomodulatory Function of Polyvinylpyrrolidone (PVP)-Functionalized Gold Nanoparticles in *Vibrio*-Stimulated Sea Urchin Immune Cells

Andi Alijagic [1], Angela Bonura [1], Francesco Barbero [2], Victor F. Puntes [2,3,4], Francesco Gervasi [5] and Annalisa Pinsino [1,*]

[1] Consiglio Nazionale delle Ricerche, Istituto per la Ricerca e l'Innovazione Biomedica (IRIB), 90146 Palermo, Italy; andialijagic@gmail.com (A.A.); angela.bonura@irib.cnr.it (A.B.)
[2] Catalan Institute of Nanoscience and Nanotechnology (ICN2), CSIC and BIST, Campus UAB, Bellaterra, 08193 Barcelona, Spain; fra.barbero@gmail.com (F.B.); victor.puntes.icn@gmail.com (V.F.P.)
[3] Institució Catalana de Recerca i Estudis Avançats (ICREA), 08010 Barcelona, Spain
[4] Vall d Hebron Institut de Recerca (VHIR), 08035 Barcelona, Spain
[5] Specialistic Oncology Laboratory Unit, ARNAS Hospitals Civico Di Cristina e Benfratelli, 90127 Palermo, Italy; francesco.gervasi@arnascivico.it
* Correspondence: annalisa.pinsino@irib.cnr.it

Table S1. Results from two-parameter flow cytometric analysis of immune cells of four sea urchin *Paracentrotus lividus*.

Donors	Subset of cells	Control	PVP-AuNPs	*Vibrio*	PVP-AuNPs *plus Vibrio*
A	Group 1	74.1	72.9	65.4	69.2
B		68.3	69.1	71.3	67.5
C		53	53.6	52.5	65.1
D		66.4	68.8	72.2	72.3
	Mean ± SD	65.5 ± 8.9	66.1 ± 8.5	65.4 ± 9.1	68.5 ± 3.0
A	Group 2	9.9	7.71	9.91	9.79
B		5.57	5.81	8.29	11.8
C		6.25	6.45	12.3	8.48
D		8.45	7.23	7.10	6.79
	Mean ± SD	7.5 ± 2.0	6.8 ± 0.8	9.4 ± 2.3	9.2 ± 2.1
A	Group 3	2.3	4.79	1.44	2.91
B		12.4	12.7	9.65	11.6
C		32	32.6	28.8	30
D		14.9	14.8	9.62	10.2
	Mean ± SD	15.4 ± 12.3	16 ± 11.3	12.4 ± 11.6	13.7 ± 11.5

A: Immunologically quiescent donor (Results are graphically represented in the Figure 4 of this manuscript). B: Immunologically quiescent donor. C: Immunologically active donor. D: Immunologically active donor.

Table S2. Individual values and the means ± standard deviations of the percentage of cells stained with Anti-CD45 and -CD14 from grouped quiescent and active *P. lividus* donors.

Donors	Percentage of cells	Control	PVP-AuNPs	*Vibrio*	PVP-AuNPs *plus Vibrio*
A	$CD45^+$	17.83	14.53	2.06	4.02
B		1.08	1.54	0.48	1.16
C		0.06	0.16	1.31	1.95
D		2.44	4.25	0.28	0.73
	Mean ± SD	5.35 ± 8.38	5.12 ± 6.5	1.03 ± 0.82	1.97 ± 1.46
A	$CD14^+$	20.43	32.03	6.41	12.47
B		2.43	3.28	1.36	3.23
C		0.03	0.05	0.16	0.32
D		1.15	0.43	0.13	0.18
	Mean ± SD	6.01 ± 9.66	8.95 ± 15.46	2.02 ± 2.99	4.05 ± 5.79

A: Immunologically quiescent donor. B: Immunologically quiescent donor. C: Immunologically active donor. D: Immunologically active donor.
